# Widespread haemorrhages in infants post-shunting (WHIPS): clinical features, risk factors and neuroimaging characteristics of a rare and under-recognised phenomenon

**DOI:** 10.1007/s00234-024-03418-8

**Published:** 2024-07-03

**Authors:** Rahul Lakshmanan, Fariza Abu Hassan, Shashini Dissanayake, Harriet Crabtree, Aden McLaughlin, Matthew Cooper, Sharon Lee, Richard Warne, Peter Shipman

**Affiliations:** 1 Department of Medical Imaging, Perth Children’s Hospital, 15 Hospital Ave, Nedlands, WA 6009 Australia; 2 Centre for Neuromuscular and Neurological disorders (Perron Institute), University of Western Australia, Perth, WA Australia; 3 Department of Medical Imaging, Fiona Stanley Hospital, Perth, WA Australia; 4 Department of Paediatrics, Perth Children’s Hospital, Perth, WA Australia; 5 Telethon Kids Institute, University of Western Australia, Perth, WA Australia; 6 Department of Neurosurgery, Perth Children’s Hospital, Perth, WA Australia

**Keywords:** Paediatric neuroradiology, Stroke, Haemorrhage, Hydrocephalus, CSF shunting, Infants, Neonates

## Abstract

**Purpose:**

Infants undergoing CSF shunting procedures face a rare complication which we propose to rename “Widespread Haemorrhages in Infants Post-Shunting” (WHIPS) to better capture this unique phenomenon specific to infants undergoing CSF diversion. Our objective is to analyse the risk factors for WHIPS development and provide a detailed neuroradiological description of these haemorrhages.

**Materials and methods:**

A radiology information system (RIS) was searched using the search terms “shunt” and/or “catheter” and/or “drain” and/or “ventriculoperitoneal” and/or “VP” between September 2008 to January 2021 for patients < 12 months of age. Clinical data was compiled for each patient meeting the inclusion criteria. Included cases were reviewed by three radiologists for the presence of WHIPS with calculation of the bifrontal ratio and documenting haemorrhage number, morphology, location and lobar distribution.

**Results:**

51 patients met inclusion criteria, 8 WHIPS patients and 43 controls. There was a statistically significant correlation between a larger post-op head circumference and WHIPS (*p* = 0.04). WHIPS was associated with post-haemorrhagic hydrocephalus and post-infectious hydrocephalus (*p* = 0.009). WHIPS were identified in the cortico-subcortical regions, periventricular white matter, and deep white matter. Haemorrhages were either punctate, ovoid or confluent. Haemorrhages ranged from single to innumerable.

**Conclusions:**

WHIPS represent a rare and under-recognised complication of CSF shunting unique to the infantile population. We postulate deep and superficial medullary venous haemorrhage as an underlying mechanism related to disordered intracranial hydrodynamics which are exacerbated in the infantile population due to underdeveloped arachnoid granulations and a compliant skull.

## Introduction

Hydrocephalus can be defined as active distention of the ventricular system resulting from inadequate passage of CSF from its point of production in the ventricles to its point of absorption to the systemic circulation [1]. There are a myriad of causes for infantile hydrocephalus broadly categorised into congenital (including genetic) and acquired disorders. Common congenital causes include myelomeningocele, aqueductal stenosis and posterior fossa malformations such as the Dandy-Walker malformation. Acquired infantile hydrocephalus in developed nations is usually the result of intraventricular haemorrhage, however globally infection is the leading cause [2]. The prevalence of infantile hydrocephalus is estimated at between 1 and 32 per 10,000 births [1] and most recently at 1.1 per 1000 births [3]. There are approximately 400,000 cases of infantile and childhood hydrocephalus worldwide annually [2]. 

Untreated, hydrocephalus in the infantile period is associated with poor neurodevelopmental outcomes and carries an increased mortality risk [2]. Infantile hydrocephalus has a high incidence of surgical treatment, reported at up to 60% [4]. CSF shunting is the mainstay of treatment for hydrocephalus, this can be in the form of temporizing procedures such as external ventricular drainage (EVD) or longer term treatments such as ventriculoperitoneal (VP) shunting. These are critical therapies and later surgical intervention for infantile acquired hydrocephalus is associated with increased mortality and adverse neurodevelopmental outcome including a Bayley Scale of Infant and Toddler Development III score < 70, cerebral palsy, epilepsy, visual and hearing impairment [5]. 

Despite the clear benefit of CSF diversion therapy for infantile hydrocephalus there are a number of potential complications which can result from the therapy itself, including well known complications of catheter associated intracerebral/ intraventricular haemorrhage, catheter malposition and later on shunt malfunction/ migration, infection, CSF over-drainage and intra-abdominal complications [6]. A lesser known complication of CSF diversion, unique to the infantile age group, has been termed “multifocal intraparenchymal haemorrhages (MIPH)” which are haemorrhages in the brain remote to the site of catheter insertion, first described by Choi et al. in 2014 [7]. We propose an alternative name “Widespread Haemorrhages in Infants Post-Shunting” (WHIPS) for this phenomenon, as the reported cases have all occurred in the infantile population [7–9], are widespread and are associated with CSF shunting, all of which are key components of this unique neuroradiological phenomenon. Due to the rarity of this phenomenon all prior studies are limited by small case numbers, however some risk factors for development of WHIPS include preterm delivery [7] (although this was not a risk factor in the study by Oushy et. al [8]), pre-operative fronto-occipital horn ratio (FOR), the change in FOR [8] and reduction in ventricular size after multiple serial ventricular reservoir taps [9]. We have studied the risk factors for development of WHIPS in our local cohort adding to the current limited body of literature and provided a more detailed assessment of the neuroimaging characteristics of these haemorrhages.

## Materials and methods

The institutional review board at the Child and Adolescent Health Service Western Australia approved this study and waived the need for formal ethics approval.

To identify infants who had undergone CSF diversion therapy using an external ventricular drain (EVD) or ventriculoperitoneal (VP) shunt a radiology information system (RIS) at two tertiary paediatric hospitals and one tertiary obstetric hospital was retrospectively searched between the period September 2008 to January 2021 using the keywords “shunt” and/or “catheter” and/or “drain” and/or “ventriculoperitoneal” and/or “VP” and limited to patients under 12 months of age. Inclusion criteria were; age < 12 months at the time of CSF diversion therapy; hydrocephalus on imaging; pre-operative and post-operative cross-sectional imaging (CT and/ or MRI); blood sensitive sequences (e.g. SWI or T2*) for those who underwent MRI. Exclusion criteria for the study were those who underwent other neurosurgical procedures (e.g. endoscopic third ventriculostomy), abnormal coagulation profiles at the time of shunting, anti-coagulant or anti-platelet medications at the time of shunting and insufficient clinical data.

The following information was recorded for each of the included patients; gender; gestational age at birth; corrected age at shunting; time to shunting; pre-operative head circumference; post-operative head circumference; head circumference % change; bifrontal ratio (BFR) pre-operative; BFR post-operative; BFR % change. The type of shunt (programmable vs. non-programmable) and reason for shunting was also recorded.

Images were reviewed by three separate radiologists including a paediatric neuroradiologist with 7 years of experience (R.L.), a paediatric radiologist with 5 years of experience (F.A.) and a radiology trainee (S.D.) for the presence of WHIPS, disagreements were resolved by consensus. WHIPS was defined as 1 or more new haemorrhage located remote to the site of CSF diversion catheter insertion, not intraventricular/ ependymal in location, not related to an underlying coagulopathy and the patient should not have had any other neurological interventions between CSF diversion catheter insertion and identification of a new haemorrhage. The bifrontal diameter was measured on cross sectional imaging for all cases pre- and post-operatively. For the WHIPS cases the time to detection of white matter haemorrhages was also recorded. All positive WHIPS cases were also reviewed in detail and the number of haemorrhages, haemorrhage morphology, haemorrhage location and lobar distribution of haemorrhages was recorded.

Statistical analysis was performed using software R (R Core Team 2021), via the RStudio IDE (RStudio Team 2016) and was compiled in RMarkdown [10]. Pearson’s Chi-squared test was applied to categorical variables and the Student’s t-test was used for continuous data, for comparing demographic and clinico-radiological characteristics between the WHIPS and control groups.

## Results

### Study population

**Fig. 1:**
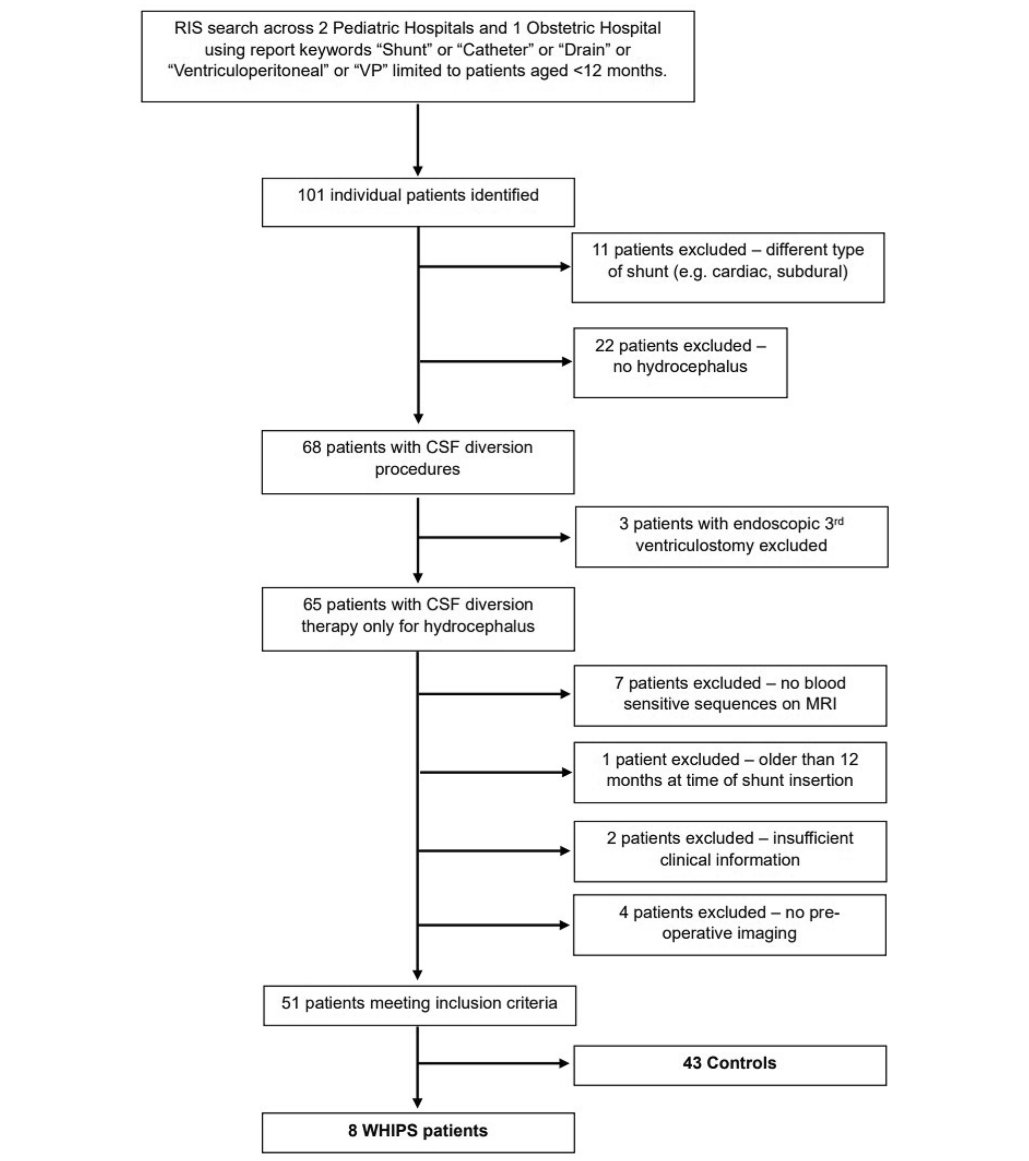
Study population flow chart

A total of 101 infants were identified though the RIS keyword search performed at two tertiary paediatric hospitals and one tertiary obstetric hospital from 2008 to January 2021. 11 patients were excluded due to no CSF diversion shunt and 22 were excluded due to the absence of hydrocephalus on imaging leaving a total of 68 patients who underwent CSF diversion for hydrocephalus. 3 patients were excluded due to the co-existence of a 3rd ventriculostomy. 7 further patients were excluded due to no blood sensitive sequences on MRI, 1 patient was excluded due to being older than 12 months of age at the time of shunting, 2 patients were excluded due to insufficient clinical information and 4 patients were excluded due to inadequate pre-operative imaging. For the remaining 51 patients, consensus neuroimaging review by three radiologists revealed 8 WHIPS patients (16%) and 43 controls (84%). The patient flow chart is shown in Fig. 1.

### Demographic and clinico-radiological characteristics

A comparison of the demographic and clinico-radiological characteristics between the WHIPS group and control group is presented in Table 1. There were no significant differences in gender, gestational age at birth, age at shunting, time to shunting, pre-op head circumference, % change in head circumference, BFR pre-op, BFR post-op or BFR % change between the two groups. There was a statistically significant difference between post-op head circumference between the WHIPS and control group, the WHIPS group having a larger head circumference post op compared to the control group (42.5 cm vs. 37.7 cm *p* = 0.04). Although not statistically significant, the WHIPS group had a lower gestational age (33.6 weeks vs. 36.7 weeks *p* = 0.34), older age at shunting (73 days vs. 58 days *p* = 0.6), longer time to shunting (18.4 days vs. 7.5 days *p* = 0.2), larger pre-op head circumference (42.7 cm vs. 38.3 cm *p* = 0.12), larger head circumference % change (3.2% vs. 1.1% *p* = 0.2), smaller pre-op BFR (0.45 vs. 0.51 *p* = 0.22) and a smaller BFR % change (-4.61% vs. -12.29% *p* = 0.24). WHIPS cases all had normal coagulation profiles and were not on any anticoagulant or anti-platelet medications at the time of shunting.

**Table 1:** WHIPS vs. controls demographic and clinico-radiological characteristics

	WHIPS	Controls	*P*.Value
N	8	43	
Gender (Female), n (%)	3 (37.5%)	16 (37.2%)	1
Gender (Male), *n* (%)	5 (62.5%)	27 (62.8%)	1
Gestational Age (weeks), mean (SD) [*n*]	33.6 (6.4) [8]	36.7 (4.1) [37]	0.34
Age at shunt (days), mean (SD) [*n*]	73.0 (69.8) [8]	58.4 (64.7) [41]	0.6
Time to shunt (days), mean (SD) [8]	18.4 (21.7) [8]	7.5 (9.1) [40]	0.2
Head circumference pre-op (cm), mean (SD) [*n*]	42.7 (3.2) [3]	38.3 (4.0) [29]	0.12
Head circumference post-op (cm), mean (SD) [*n*]	42.5 (2.3) [3]	37.7 (3.2) [24]	0.04
Head circumference % change, mean (SD) [*n*]	3.2 (1.3) [2]	1.1 (5.3) [22]	0.2
BFR pre-op, mean (SD) [*n*]	0.45 (0.11) [8]	0.51 (0.10) [41]	0.22
BFR post-op, mean (SD) [*n*]	0.44 (0.15) [8]	0.44 (0.09) [41]	0.96
BFR % change, mean (SD) [*n*]	-4.61 (15.73) [8]	-12.29 (16.37) [41]	0.24

### Shunt types and indications

The shunt types and indications between the WHIPS and control groups are compared in Table 2. There was no statistically significant difference between the two groups in terms of type of shunt used (programmable vs. non-programmable, *p* = 0.29). A higher proportion of non-programmable shunts was used in the WHIPS group (62.5%) vs. the control group (34.1%). Comparison of the indications for shunting demonstrated a statistically significant difference, the WHIPS group more likely (*p* = 0.009) to be shunted for intraventricular haemorrhage (IVH) (62.5%) and infection (37.5%) compared to the control group (42.9% IVH and 2.4% infection).

**Table 2:** WHIPS vs. control shunt types and shunt indications

	WHIPS	Control
**Type of Shunt**		
Non programmable, *n* (%)	5 (62.5%)	14 (34.1%)
Programmable, *n* (%)	3 (37.5%)	24 (58.5%)
Not documented *n* (%)	0 (0.0%)	3 (7.3%)
**Reason for shunt**		
Mass *n* (%)	0 (0.0%)	10 (23.8%)
Craniosynostosis *n* (%)	0 (0.0%)	1 (2.4%)
IVH *n* (%)	5 (62.5%)	18 (42.9%)
Chiari II malformation *n* (%)	0 (0.0%)	10 (23.8%)
Congenital Aqueductal stenosis *n* (%)	0 (0.0%)	2 (4.8%)
Infection *n* (%)	3 (37.5%)	1 (2.4%)

### WHIPS neuroimaging characteristics

**Fig. 2:**
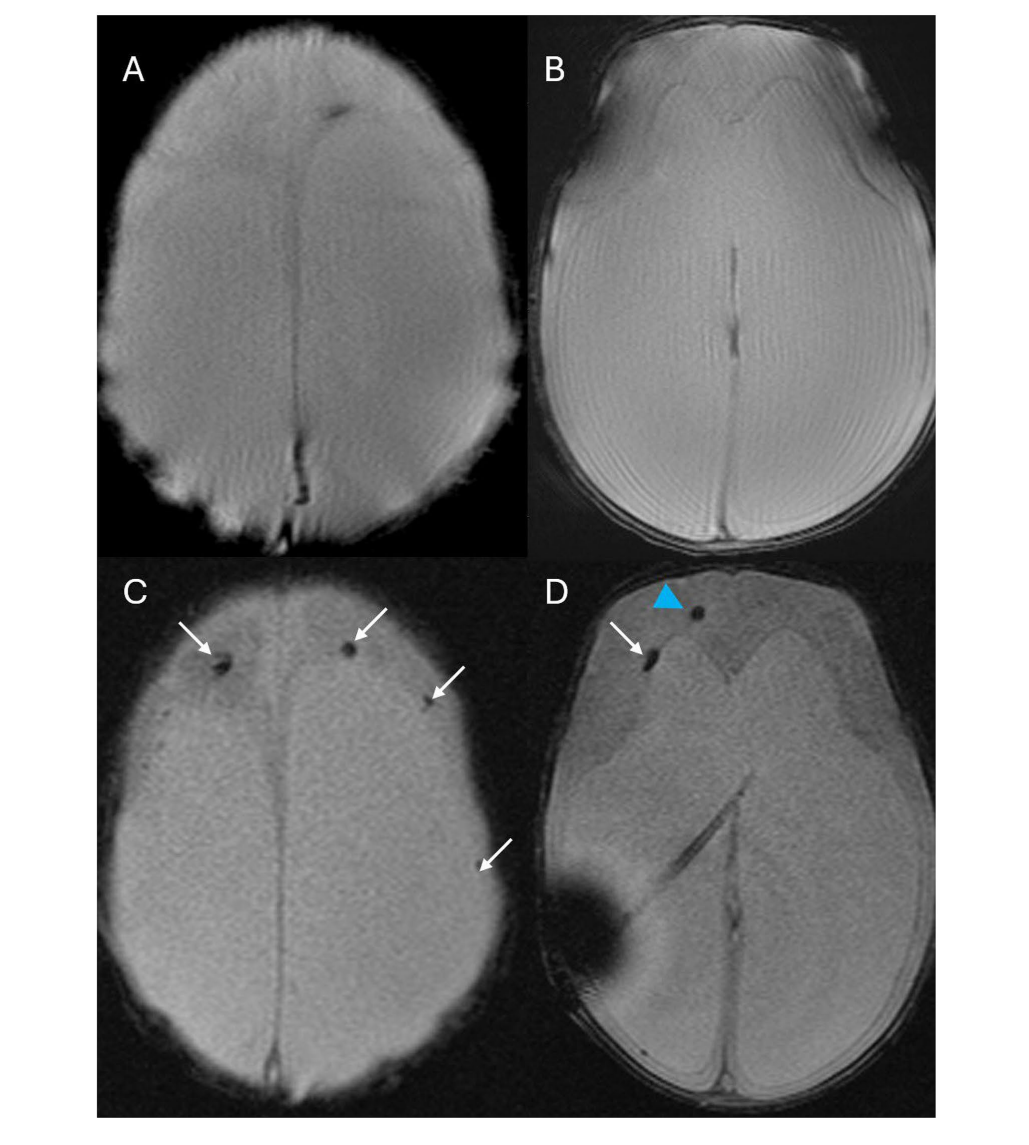
**(a-b)** Axial T2* imaging pre-shunting at 24 days demonstrating severe post-haemorrhagic hydrocephalus. **(c-d)** Axial T2* imaging at 87 days of age (29 days post right transparietal VP shunt insertion) demonstrating bilateral frontal periventricular (white arrows) and deep white matter (blue arrowhead) ovoid WHIPS

**Fig. 3:**
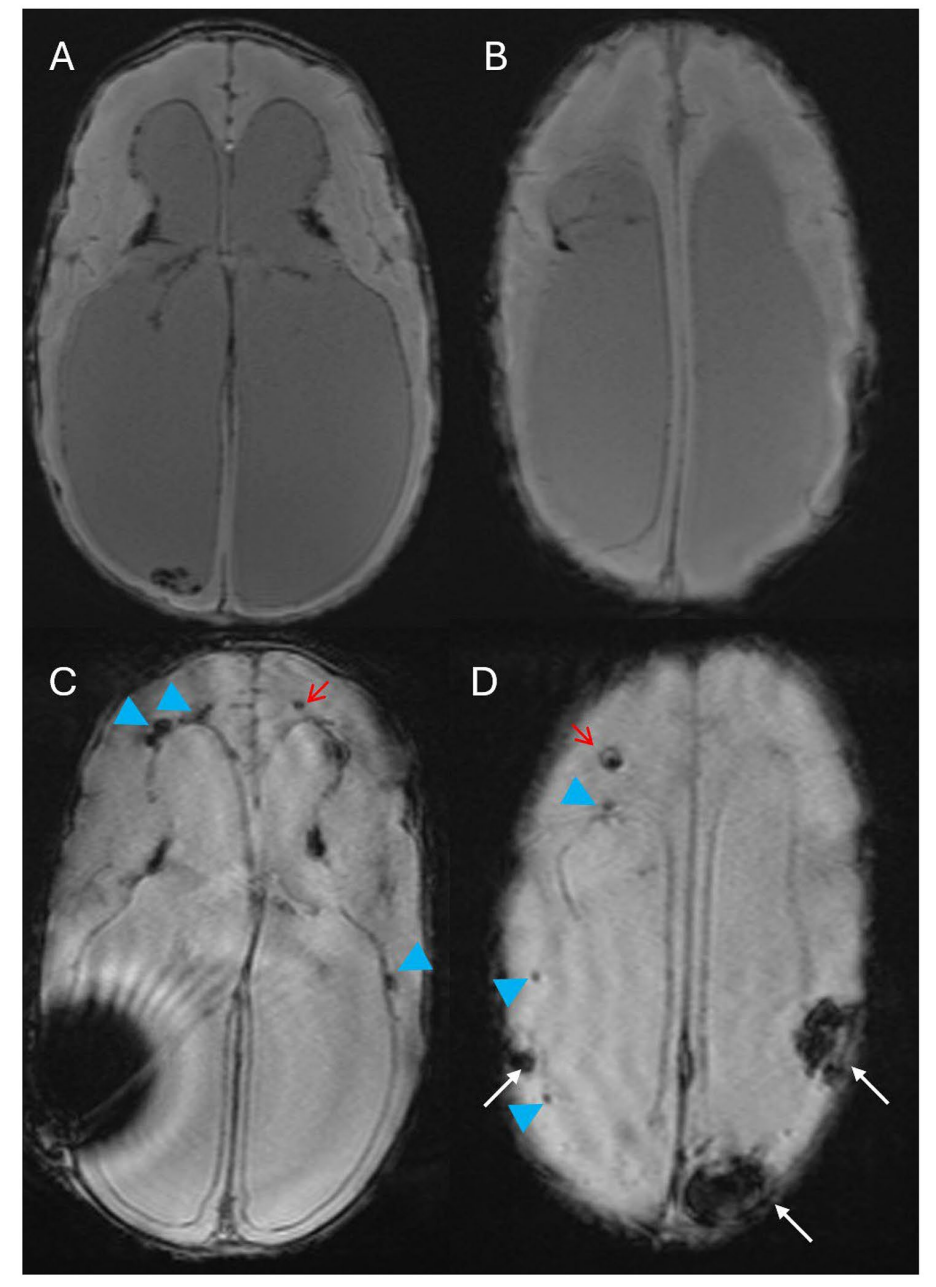
**(a-b)** Axial SWI performed before shunting in a 41 day old neonate with post-haemorrhagic hydrocephalus. **(c-d)** Axial T2* performed 11 days post right transparietal VP shunt insertion demonstrating multiple punctate and ovoid WHIPS involving the periventricular (blue arrow heads) and deep white matter (red arrows). Cortico-subcortical haemorrhages are also present in d (white arrows), the largest of these in the left posterior frontal and left medial parietal lobe

**Fig. 4:**
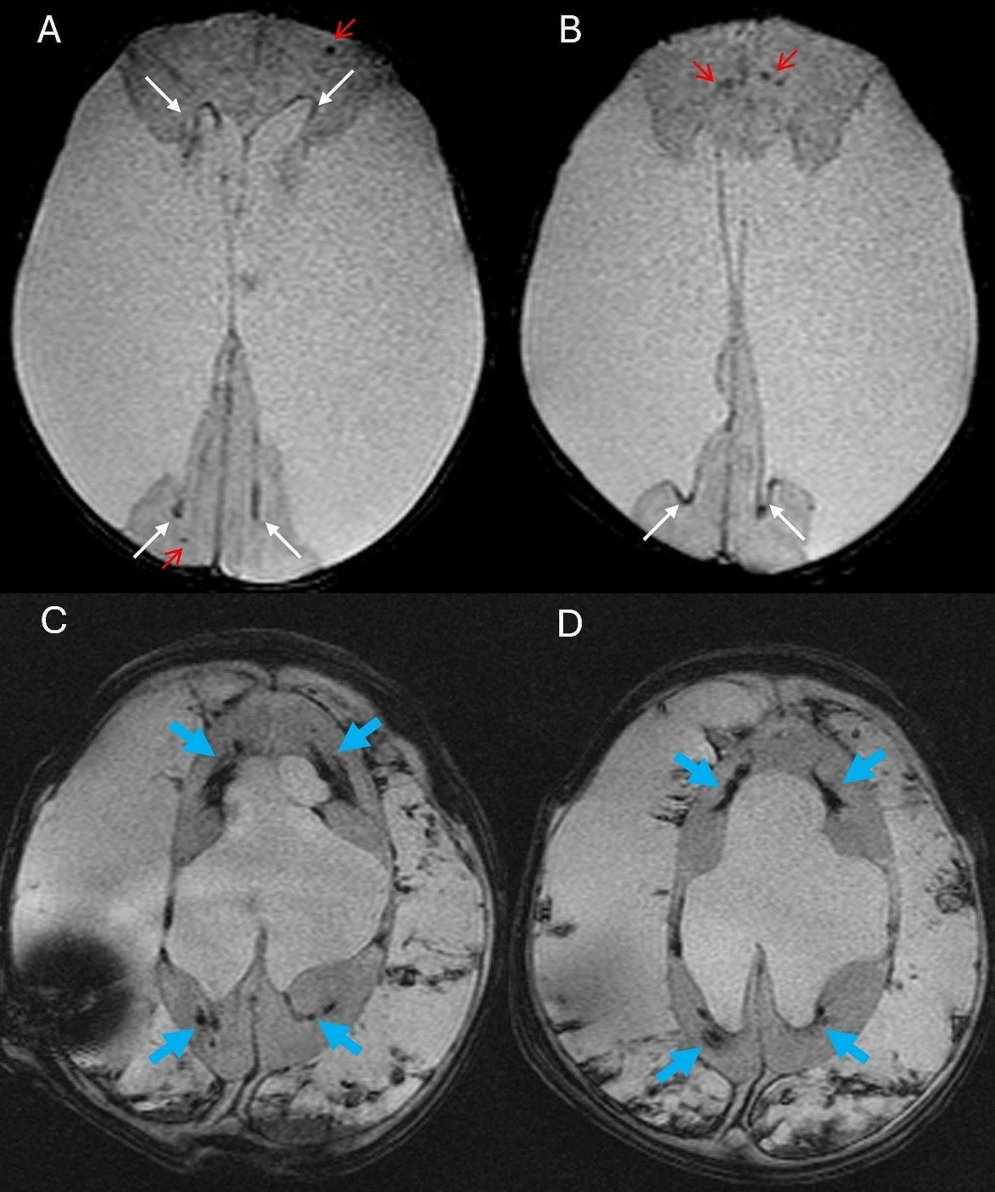
**(a-b)** Axial T2* imaging pre-shunting in a 3 day old with a COL4A1 mutation demonstrating post-haemorrhagic hydrocephalus in communication with bilateral large porencephalic cysts. Some areas of susceptibility are seen along the ependymal margins of the ventricles (white arrows) and in the frontal and occipital white matter (red arrows), which are shown to represent calcifications on CT (see Fig. 5), a finding related to the underlying COL4A1 mutation. **(c-d)** Axial T2* MRI at 3 months of age 13 days after insertion of a right transparietal VP shunt showing extensive confluent bilateral fan-shaped periventricular and deep white matter WHIPS (blue arrows) similar to the “iris sign” in deep medullary vein haemorrhage, these are most prominent in the frontal and parietal regions. There are also large bilateral post-operative haemorrhagic subdural collections with worsened hydrocephalus

**Fig. 5:**
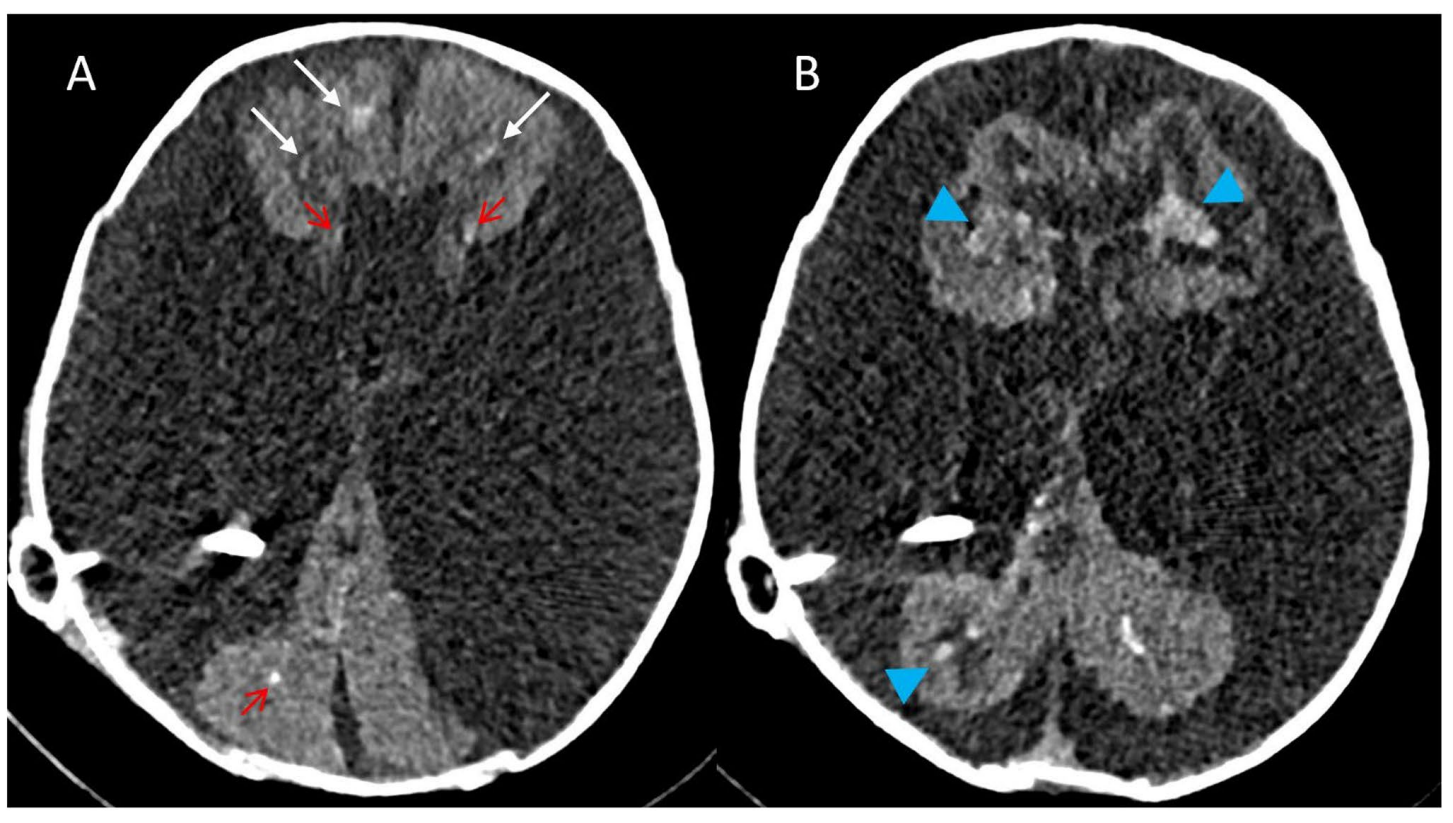
**(a)** Axial CT performed day 2 post right transparietal VP shunting for post-haemorrhagic hydrocephalus and porencephalic cysts due to an underlying COL4A1 mutation (same patient as Fig. 4) shows bilateral frontal WHIPS in A (white arrows) with evidence of ependymal and white matter calcifications (red arrows). **(b)** Axial CT performed 11 days later shows progression of bilateral frontal WHIPS and new right occipital WHIPS (blue arrowheads) surrounded by progressive low attenuation oedema. Bilateral enlarging CSF attenuation subdural collections are also evident in B distorting the parenchyma

Three WHIPS patients had pre- and post-operative T2* MRI, two had pre- and post-operative SWI MRI and three had pre- and post-operative CT for WHIPS identification. WHIPS varied greatly in number ranging from 1 to innumerable haemorrhages. WHIPS were identified in the periventricular white matter in 4/8 cases (Fig. 2), deep white matter in 3/8 cases (Fig. 2), cortico-subcortical region in 5/8 cases (Figs. 3) and 3/8 patients had involvement of two or more regions (Figs. 2 and 3). Ovoid lesions were seen in 5/8 cases and ranged from < 5 mm to 15 mm in size. Punctate lesions were seen in 4/8 cases and tended to be very small < 5 mm. One patient (case 5, Table 3) had innumerable severe confluent fan-shaped WHIPS, reminiscent of the “iris sign” in deep medullary venous haemorrhage (Fig. 4), however the severity of the WHIPS in this patient may have been compounded by the underlying COL4A1 mutation [11]. This same patient also demonstrated progression of WHIPS between CT studies performed day 2 and day 11 post insertion of a right trans-parietal VP shunt (Fig. 5), again this progression could have been confounded by the underlying COL4A1 mutation. In terms of lobar distribution in the brain 5/8 had involvement of the frontal lobes, 5/8 had parietal involvement, 3/8 had temporal involvement and 2/8 had occipital lobe involvement, with more than one lobe involved in 3/8 patients. The deep grey matter structures and infratentorial brain were not involved in our WHIPS cohort. The more severe the WHIPS the longer the time to shunting, the two patients with innumerable WHIPS were associated with shunting 24 and 35 days after identification of hydrocephalus and the patient with 11 WHIPS waited 63 days for CSF diversion after hydrocephalus was diagnosed. The WHIPS patients with the largest haemorrhage burden all had shunting for post-haemorrhagic hydrocephalus. This information is summarised in Table 3. In terms of other complications after shunting other than WHIPS development, 2/8 WHIPS cases had new bilateral subdural collections, notably there were no cases of subpial haemorrhage identified in the WHIPS cohort.

**Table 3:** WHIPS neuroimaging characteristics

Case No.	Hydrocephalus cause	WHIPS number	Locations	Morphology	Lobes involved	Time to shunting*
**1**	PHH-p	11	PV, DWM, CS	Ovoid < 5 mm	F	63 days
**2**	PHH-p	3	PV	Punctate	F	4 days
**3**	PHH-p	2	CS	Punctate	F, T	3 days
**4**	PHH-p	Innumerable	PV, DWM, CS	Punctate and ovoid 5–15 mm	F, T, P, O	24 days
**5**	PHH-c	Innumerable	PV, DWM	Confluent, fan-shaped	F, T, P, O	35 days
**6**	PIH-h	2	CS	Ovoid 5–10 mm	*P*	4 days
**7**	PIH-t	2	CS	Ovoid < 5 mm	*P*	0 days
**8**	PIH-t	1	DWM	Ovoid < 5 mm	*P*	14 days

PHH-p = Post-haemorrhagic hydrocephalus preterm; PHH-c = Post-Haemorrhagic Hydrocephalus due to COL4A1 mutation; PIH-h = Post-Infectious Hydrocephalus due to *H. Influenza*; PIH-t = Post-Infectious Hydrocephalus due to congenital *Toxoplasma Gondii* infection; PV = Periventricular; DWM = Deep White Matter; CS = Cortico-Subcortical; F = Frontal; T = Temporal; P = Parietal; O = Occipital. *Time to shunting indicates the time from first identification of hydrocephalus on imaging to institution of CSF diversion therapy

## Discussion

We advocate for a modification to the previously established term “Multifocal Intra-Parenchymal Haemorrhages” (MIPH), which describes a rare complication of CSF diversion therapy in infants. The existing name fails to encompass crucial aspects of this neuroradiological phenomenon, observed exclusively in the infantile population following CSF shunting. The proposed term “Widespread Haemorrhages in Infants Post-Shunting” (WHIPS) more precisely captures the essence of this unique clinico-radiological entity. The intention behind this change is to enhance awareness of the phenomenon as a potential complication of shunting in the infantile population.

In our cohort, WHIPS exhibited an incidence of 16%, a rate notably higher than the reported 2.9% in infants by Choi et al. [7]. and 9.1% in neonates (age < 30 days) as per Oushy et al.‘s study [8]. This heightened incidence in our study may stem from more rigorous exclusion criteria. Our criteria involved the exclusion of patients lacking pre-operative imaging, those without blood-sensitive sequences on pre- or post-operative MRI, those who underwent 3rd ventriculostomy and those with insufficient clinical information. Consequently, our cohort comprised 51 eligible patients over a 12-year 4-month period (September 2008 to January 2021), in contrast to Oushy et al.‘s study, which included 121 patients over a 17-year span [8] and Choi et al.‘s study, encompassing 140 infantile patients over an 11-year duration [7]. One additional case series by Okazaki et al. reported multiple new “germinal matrix haemorrhages” after shunting in two term born neonates with congenital hydrocephalus one as a result of craniosynostosis and another secondary to a complicated interhemispheric/ arachnoid cyst [12], the images from this paper demonstrate what appear to be multiple periventricular WHIPS in both these patients, likely erroneously labelled as recurrent germinal matrix haemorrhage.

Our study revealed a statistically significant positive correlation between WHIPS and a larger post-operative head circumference. Possibly as a result of the limited case numbers in our cohort, no other statistically significant differences in demographic or clinic-radiological characteristics were identified. It is worth noting, however, that the WHIPS group exhibited a lower mean gestational age, older mean age at shunting, longer mean time to shunting, larger mean head circumference, a larger pre-operative head circumference, larger head circumference % change, smaller pre-op BFR, and smaller BFR % change. Additionally, in our cohort, the WHIPS group exhibited a statistically significant association with post-haemorrhagic and post-infectious hydrocephalus compared to the control group. In Choi et al.‘s study, statistically significant associations were reported between WHIPS and corrected age < 3 months, preterm birth, absence of prior intracranial surgery, and fronto-occipital ratio [7]. Oushy et al. identified statistically significant associations between WHIPS and the etiology of hydrocephalus (aqueductal stenosis most common), type of CSF diversion (VP shunt most common), and shunt valve classification (flow control most common) [8]. Cizmeci et al. explored WHIPS in the context of serial ventricular reservoir taps in neonates, revealing statistically significant associations between WHIPS and reductions in ventricular index and thalamo-occipital distance 7 days after reservoir tap [9]. 

We have expanded the neuroradiological characterization of WHIPS, revealing that these haemorrhages exhibit a spectrum from being singular to innumerable, distributed throughout the supratentorial brain parenchyma—from the periventricular white matter and deep white matter to the cortico-subcortical regions. Our findings indicate WHIPS involvement occurs across all lobes of the supratentorial brain, with noteworthy sparing of the deep grey matter structures and infratentorial brain in our studied cohort. In one of our WHIPS cases, the haemorrhages displayed a fan-shaped confluent and diffuse pattern, reminiscent of the “iris sign” observed in deep medullary venous haemorrhage, this case also showed large post-operative bilateral subdural haemorrhages [11]. This “iris sign” was also seen in Figs. 2 and 3 of the original description of this entity by Choi et al. [7], both of these reported cases also demonstrated bilateral subdural collections after shunting. Notably, our patient with the “iris sign” also illustrated the progressive nature of these haemorrhages, with a significant increase in haemorrhage burden over a 9-day period (see Fig. 5), a facet not previously detailed in descriptions of this entity. Furthermore, our observations reveal that more extensive WHIPS cases in our cohort were associated with a longer duration between the initial identification of hydrocephalus and the commencement of shunting, all occurring in patients with post-haemorrhagic hydrocephalus.

In terms of prognosis, previous studies have reported negative associations with cognitive and motor outcomes at 2 years of age [9]. A higher rate of shunt revision within a year of initial CSF diversion was noted in the study by Oushy et al. [8]. No long term follow up data was available in our study nor the study by Choi et al. [7]

The underlying mechanisms of WHIPS remain somewhat elusive. However, the complex interplay among CSF flow dynamics, potential rapid fluctuations in CSF volume and pressure, and the susceptibility of the infantile brain to both deep medullary and superficial medullary venous injuries may contribute to this complication arising from shunting in infants [11]. Rekate’s model of intracranial hydrodynamics [13–15] sheds light on the interaction between CSF circulation and the cerebral microvasculature, particularly the capillaries and venules within the cerebral parenchyma, which are involved in CSF absorption.

The unique developmental characteristics of the infantile population further complicate the picture. The underdeveloped arachnoid granulations, with little role in CSF absorption in infants, likely lead to an increased reliance on the cerebral venous system for CSF balance. Additionally, the infantile head’s elastic nature, owing to the non-closure of sutures, normally enables swift changes in intracranial volumes in response to changes in CSF volume. When there is loss of the normal elastic response of the infantile skull to a rapid reduction in intracranial CSF volume after shunting, there may be an increased reliance on the venous circulation to compensate for the reduced intracranial pressure by increasing venous pressures, this may in turn precipitate venous haemorrhages within the superficial and deep medullary venous circulation [4]. Our statistically significant finding of an increased post-shunting head circumference in the WHIPS group supports this hypothesis.

The pathophysiology of WHIPS may also involve the swift restoration of cerebral parenchymal venous absorption in both the deep and superficial medullary veins after CSF diversion. This venous hypothesis aligns with the presence of the neuroimaging “iris sign” in one of our cases and in some case figures documented in the literature [7], a pathognomonic feature of infantile cerebral parenchymal haemorrhage originating from the deep medullary venous system [11]. The cortico-subcortical haemorrhages observed in our WHIPS cohort might indicate bleeding originating from the superficial cerebral venous system, responsible for draining much of the cortex and subcortical white matter [11]. Disordered cerebral autoregulation and mechanical vascular injury resulting from changes in intracranial pressure as well as neuroinflammation could also contribute to the development of WHIPS [8, 16]. 

Our study is subject to several limitations. These include the retrospective study design, a small number of cases, and the use of heterogeneous imaging modalities both pre- and post-operatively. This variability in imaging techniques, with CT being less sensitive than T2* and T2* less sensitive than SWI, may have impacted the detection sensitivity for new haemorrhages, with potential underestimation of haemorrhage burden in those who only underwent CT or T2* post-operatively. During the analysis of cases for the presence of WHIPS, distinguishing blooming artefact from new shunt related IVH from periventricular WHIPS proved challenging in certain instances. Additionally, artefact from the shunt catheters may have obscured new WHIPS in some patients, potentially leading to a reduction in case numbers. Notably, our study did not explore the long-term neurodevelopmental outcomes of infants with WHIPS. Given the severe underlying disorders causing hydrocephalus, such as post-haemorrhagic hydrocephalus and post-infectious hydrocephalus, it would however be challenging to isolate the contribution of WHIPS alone to overall neurodisability compared to neurodisability resulting from the background disease process.

## Conclusions

WHIPS represents a potentially under-recognised complication specific to the infantile population following CSF shunting. In contrast to the previously utilized term MIPH, we advocate for the name WHIPS, as it more accurately captures the uniqueness of this disorder in infants who have undergone CSF shunting. In our cohort, we observed a higher incidence of WHIPS (16%) compared to the reported rates in other studies (2.9 − 9.1%) [7, 8]. Notably, we identified a statistically significant association between WHIPS and an increased post-shunting head circumference. Our study offers an in-depth neuroradiological characterization of WHIPS, a dimension that has not been thoroughly investigated in prior research. The neuroimaging features of WHIPS strongly suggest a venous aetiology for the widespread haemorrhages, particularly highlighted by the presence of the “iris sign” observed in a case within our cohort and documented cases in the literature. The sole study examining neurodevelopmental follow-up in WHIPS, limited to neonates undergoing repeated CSF drainage via reservoir, revealed a negative association with cognitive and motor outcomes at 2 years [9]. However, longer-term studies are essential to fully grasp the neurodevelopmental implications of WHIPS.

## Abbreviations

MIPH: Multifocal Intraparenchymal Haemorrhages
WHIPS: Widespread Haemorrhages in Infants Post-Shunting
RIS: Radiology information system
EVD: External Ventricular Drain
VP: Ventriculoperitoneal
IVH: Intraventricular Haemorrhage
FOR: Fronto-occipital horn ratio
BFR: Bifrontal ratio
APTT: Activated partial thromboplastin time
INR: International normalised ratio
